# Flammenfiguren – Eosinophile, die die Haut in Brand setzen

**DOI:** 10.1007/s00105-023-05216-4

**Published:** 2023-09-20

**Authors:** Lea-Sophie Stahl, Ann-Christin Wolters, Ocko Kautz, Nikolaos Patsinakidis, Ulrike Raap

**Affiliations:** 1 Abteilung für experimentelle Allergologie und Immundermatologie, Carl von Ossietzky Universität Oldenburg, Oldenburg, Deutschland; 2 Universitätsklinik für Dermatologie und Allergologie, Klinikum Oldenburg AöR, Rahel-Straus-Str. 10, 26133 Oldenburg, Deutschland; 3 Hautarztpraxis Achternstraße, Oldenburg, Deutschland; 4 Dermatohistopathologie NordWestHisto, Westerstede, Deutschland

**Keywords:** Wells-Syndrom, Eosinophile Zellulitis, Dapson, Dexamethason, Therapieoptionen, Wells syndrome, Eosinophilic cellulitis, Dapsone, Dexamethasone, Therapeutic options

## Abstract

Eine 50-jährige Landwirtin mit der initialen Verdachtsdiagnose eines Granuloma anulare disseminatum wurde über 10 Jahre lokal mittels topischen Glukokortikoiden sowie wiederholten Phototherapien ohne suffizienten Erfolg behandelt. Im Rahmen der Vorstellung in unserer Klinik erfolgte eine histopathologische Untersuchung mit dem Ergebnis einer eosinophilen Zellulitis mit typischen „flame figures“ in der Histologie. Durch eine Systemtherapie mit Steroidstoßtherapie zeigte sich eine Abheilung der Hautveränderungen sowie des ausgeprägten Pruritus. Dieser Kasus stellt einen Bericht über eine erfolgreiche Therapie einer Patientin mit eosinophiler Zellulitis dar.

## Abstract (en)

A 50-year-old female farmer was initially diagnosed with generalized granuloma annulare and treated with local steroids and ultraviolet (UV) light therapy for 10 years, albeit without success. A histopathological examination in our clinic changed the diagnosis to Wells’ syndrome, based on the typical findings of eosinophilic cellulitis together with flame figures. A systemic approach with pulse steroid therapy resulted in complete remission of pruritus and skin manifestations. This case demonstrates successful treatment of a patient with eosinophilic cellulitis.

## Anamnese

Eine 50-jährige Landwirtin stellte sich mit seit 10 Jahren rezidivierend auftretenden multiplen erythematösen, anulär betonten, teils konfluierenden Papeln vor. Die Effloreszenzen zeigten sich in disseminierter Verteilung insbesondere stammbetont. Zudem litt die Patientin unter schwerem chronischem Pruritus. Vorerkrankungen, Allergien oder eine korrelierende Familienanamnese bestanden nicht. Aufgrund des klinischen Erscheinungsbildes und einer ambulant externen Probebiopsie wurde initial die Diagnose eines Granuloma anulare disseminatum gestellt. Wegen des atypischen Verlaufs und des inflammatorischen klinischen Aspektes entschieden wir uns für eine erneute Hautbiopsie.

## Klinischer Befund

**Figure 1:**
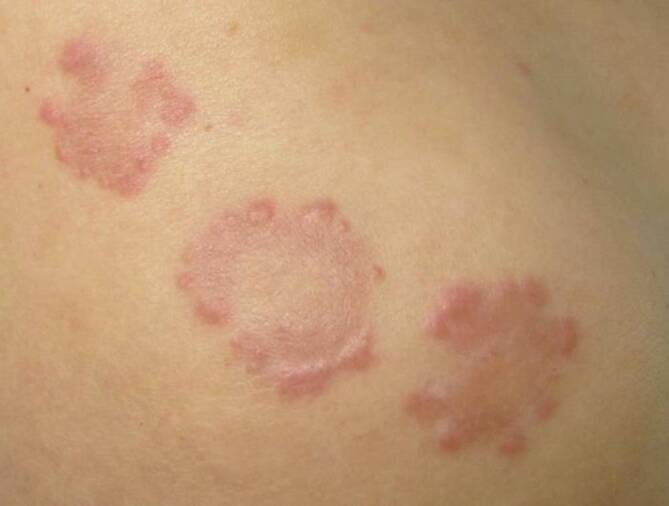

Im Rahmen der dermatologischen Untersuchung präsentierten sich am Stamm sowie den Extremitäten multiple erythematös-livide, teils konfluierende und anulär angeordnete Papeln in disseminierter Verteilung (Abb. 1). Der Pruritus wurde mit 9–10/10 auf der numerischen Rating-Skala (NRS) angegeben.

## Histopathologische Untersuchung

**Figure 2:**
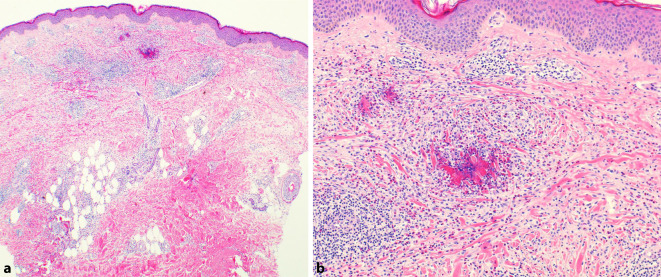

In der histologischen Untersuchung zeigten sich neben einem dichten perivasal gelagerten eosinophilenreichen Infiltrat auch sog. „flame figures“ (Abb. 2a, b), ein durch degranulierende eosinophile Granulozyten entstehendes histologisches Reaktionsmuster.

## Diagnostik

Leber‑/Nierenparameter, das Differenzialblutbild sowie eine Umfelddiagnostik mittels Thoraxröntgenaufnahme und Abdomensonographie zeigten keinen pathologischen Befund.

## Therapie und Verlauf

Bei fehlenden klinischen sowie laborchemischen Anzeichen einer systemischen Beteiligung, unter Berücksichtigung der großen klinischen Variabilität und der histologisch nachgewiesenen „flame figures“ stellten wir die Diagnose eines Wells-Syndroms.

**Figure 3:**
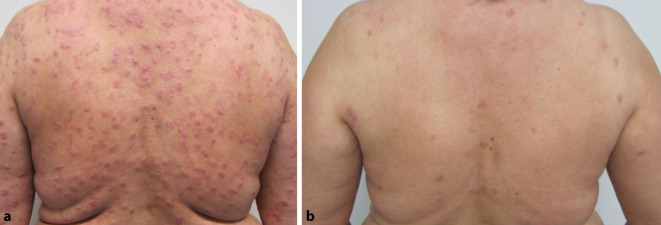

Da die Patientin über einen ausgeprägten Leidensdruck, v. a. in Form von massivem Pruritus, klagte, erfolgte zunächst eine systemische Therapie mittels Prednisolon 1 mg/kg Körpergewicht (in reduzierender Dosierung). Bei einer Prednisolon-Dosis von 7,5 mg/Tag erlitt die Patientin eine Befundexazerbation, weshalb wir die Umstellung der systemischen Therapie auf Dapson im Off-label-Use (100 mg/Tag) über insgesamt 10 Wochen initiierten. Bei beginnender Anämie (Hämoglobinwertabfall von 14,9 g/dl auf 10,1 g/dl innerhalb von 10 Wochen) wurde diese Therapie im Verlauf pausiert. Zuletzt erfolgte eine Stoßtherapie mittels Dexamethason (100 mg i.v.) über 3 Tage, die in einem Intervall von je 4 Wochen wiederholt wurde. Diese Therapie wurde nach insgesamt 7 Zyklen bei vollständiger Abheilung der Hautveränderungen und des Pruritus beendet. In den folgenden Verlaufskontrollen zeigte sich die Patientin symptomfrei (Abb. 3a, b).

## Diskussion

Das Wells-Syndrom wurde 1971 erstmals durch G. C. Wells beschrieben und stellt eine seltene autoinflammatorische Dermatose weitestgehend unbekannter Ätiologie und Pathogenese dar [1].

Klinisch weist das Wells-Syndrom in der Regel einen biphasischen Verlauf auf. Die Frühphase wird mit einem ausgeprägten Juckreiz mit Bildung von umschriebenen, scharf begrenzten stamm- und extremitätenbetonten Erythemen mit teils urtikariellen Anteilen sowie Ödem- und Blasenbildung beschrieben. Dieses erste Stadium persistiert in der Regel für wenige Tage bis Wochen. Das sekundäre Stadium ist mit einer zentralen Abblassung des Erythems assoziiert, wobei die Randbereiche einen violetten Farbton annehmen können [2–4]. In wenigen Fällen geht das Wells-Syndrom zudem mit systemischen Zeichen wie Fieber oder Arthralgien einher [4, 5]. Differenzialdiagnostisch kommen v. a. aufgrund des klinischen Erscheinungsbildes ein breites Spektrum an Erkrankungen wie Urtikaria, Granuloma anulare, Kontaktdermatitis, Erysipel sowie eine eosinophile Granulomatose mit Polyangiitis in Betracht [5].

Histopathologisch wird ein dermales Ödem mit eosinophilenreichem Infiltrat beschrieben. Aufgrund einer Eosinophilendegranulation kommt es zu einer Denaturierung von Kollagenfasern, wodurch die typischen „flame figures“ entstehen [2, 5]. Diese Flammenfiguren sind nicht spezifisch für das Wells-Syndrom, stellen jedoch ein häufiges Reaktionsmuster bei eosinophilenreichen Dermatosen dar und bestätigen die Notwendigkeit der Korrelation der histopathologischen Befunde mit dem klinischen Bild der vorliegenden Dermatose [3, 6].

Aufgrund einer beschriebenen Assoziation des Wells-Syndroms zu malignen Neoplasien, viralen Infektionen wie beispielsweise Varizellen, Diabetes mellitus oder Pilzinfektionen führten wir eine Umgebungsdiagnostik mittels laborchemischer Untersuchungen sowie bildgebender Verfahren durch [2, 7, 8], ohne dass sich ein Anhalt für eine zugrunde liegende bzw. assoziierte Erkrankung ergab.

Ein in der Literatur beschriebenes häufiges Auftreten von Rezidiven erlitt unsere Patientin seit dem erstmaligen Auftreten der Dermatose vor 10 Jahren insgesamt 2‑mal im Verlauf [2].

Da das Wells-Syndrom nicht nur eine seltene Dermatose darstellt, sondern gleichzeitig eine große klinische Polymorphie aufweist, sind Rückschlüsse bezüglich der unterschiedlichen Therapiemöglichkeiten erschwert. In der Literatur werden multiple Therapieansätze des Wells-Syndroms mit jeweils unterschiedlichen Therapieerfolgen berichtet. Empfohlene Therapieoptionen beinhalten eine Behandlung mittels Dapson, systemischer Steroide, PUVA(Psoralen plus UV-A)-Therapie und Ciclosporin [4]. In unserem Fall musste eine Therapie mit Dapson aufgrund einer Anämie beendet werden. Die Hochdosisintervalltherapie mit Dexamethason (100 mg i.v. über 3 Tage) in 4‑wöchigen Intervallen führte dann zu einer Remission (Abb. 3a, b). Neue Therapieoptionen, die derzeit noch im Off-label-Bereich liegen, umfassen die Inhibition von IL(Interleukin)-4 und IL-13 im Rahmen der Gabe von Dupilumab, das in Fallberichten erfolgreich beim Wells-Syndrom eingesetzt werden konnte [9].

Das Wells-Syndrom sollte bei therapierefraktären urtikariellen sowie blasenbildenden Dermatosen in Betracht gezogen werden und lässt sich von diesen Differenzialdiagnosen durch das klinische Erscheinungsbild in Kombination mit dem histopathologischen Nachweis der „flame figures“ abgrenzen.

## Fazit für die Praxis

- Das Wells-Syndrom stellt eine seltene inflammatorische Dermatose mit Assoziationen zu Malignomen, viralen Infekten oder Diabetes mellitus dar.
- Das Wells-Syndrom sollte als Differenzialdiagnose urtikarieller, prurigoformer Dermatosen berücksichtigt werden.
- Nach Ausschluss systemischer (Vor‑)Erkrankungen kann als suffizientes Therapieschema eine Steroidstoßtherapie erfolgen.
- Ebenfalls erwogen werden kann eine systemische Therapie („off-label“) mittels Dapson unter regelmäßiger laborchemischer Kontrolle von Blutbild und Methämoglobin (Met-Hb) oder die Gabe von Dupilumab mit Inhibition von IL(Interleukin)-4 und IL-13.
